# Supplementary material for: DUSP26 protects against acute kidney injury by dephosphorylating p53 at serine 312
Source: Nat Commun. 2026 Feb 26;17:3208. doi: 10.1038/s41467-026-69688-3 (PMC13056927; doi:10.1038/s41467-026-69688-3)
Supplement: Supplementary file 1 — Supplementary Information [file 41467_2026_69688_MOESM1_ESM.pdf]

Supplementary Materials

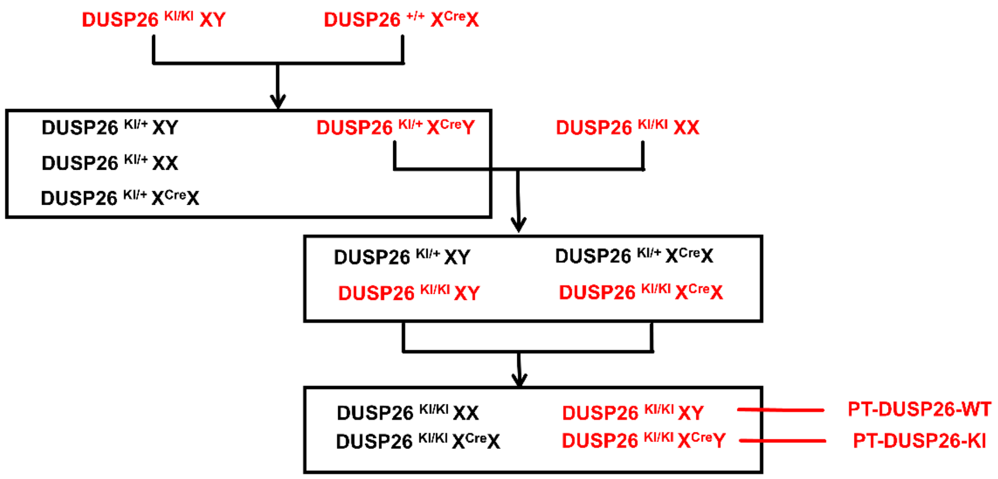

Supplementary Figure 1. Breeding protocol to generate PT-DUSP26-KI mice.

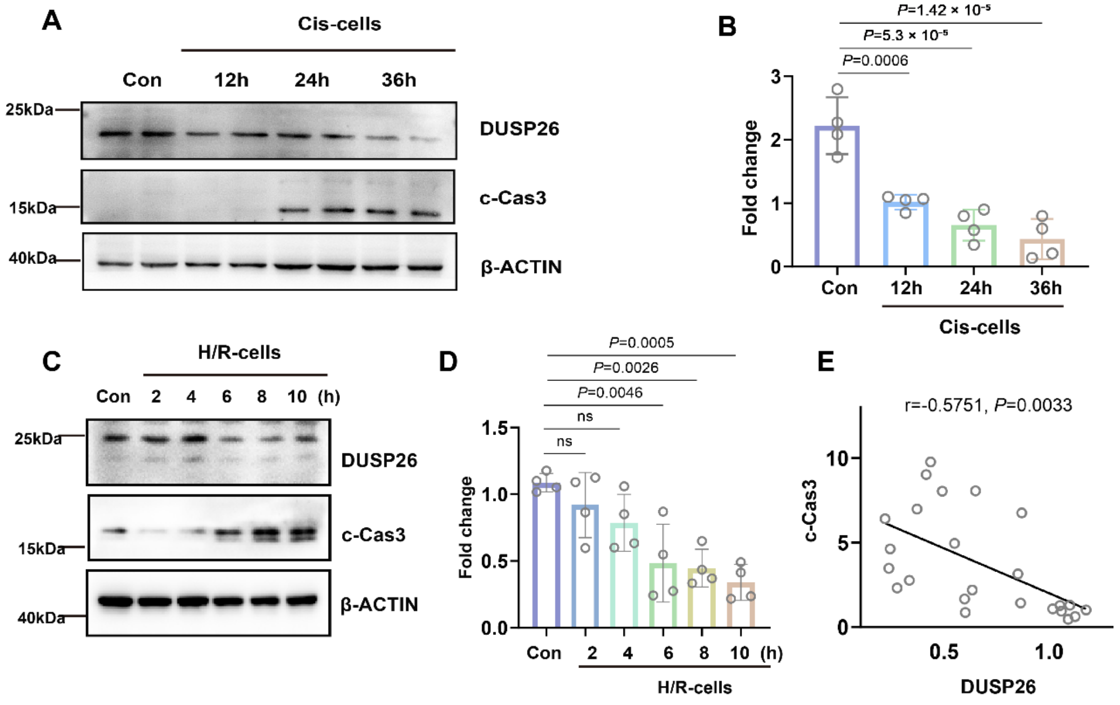

Supplementary Figure 2. DUSP26 is progressively downregulated in proximal tubular epithelial cells in response to cisplatin treatment and hypoxia/reoxygenation.

(A) Representative immunoblots of DUSP26 and cleaved caspase-3 (c-Cas3) in BUMPT cells treated with cisplatin for 12, 24, or 36 h.

(B) Densitometric quantification of DUSP26 in (A), normalized to the corresponding loading control and expressed relative to the 0 h/untreated group.

(C) Representative immunoblots of DUSP26 and c-Cas3 in BUMPT cells subjected to hypoxia/reoxygenation (H/R) for 2, 4, 6, 8, or 10 h.

(D) Densitometric quantification of DUSP26 in (C), normalized to the corresponding loading control and expressed relative to the 0 h/normoxia control.

(E) Pearson correlation (two-sided) between DUSP26 and c-Cas3 levels across H/R conditions.

n = 4 biologically independent experiments for all panels; representative blots are from experiments repeated independently with similar results. Statistical significance was assessed by one-way ANOVA with Tukey's multiple-comparisons test (two-sided; adjusted *P* values) for (B) and (D). Exact *P* values are shown in the plots. Source data are provided as a Source Data file.

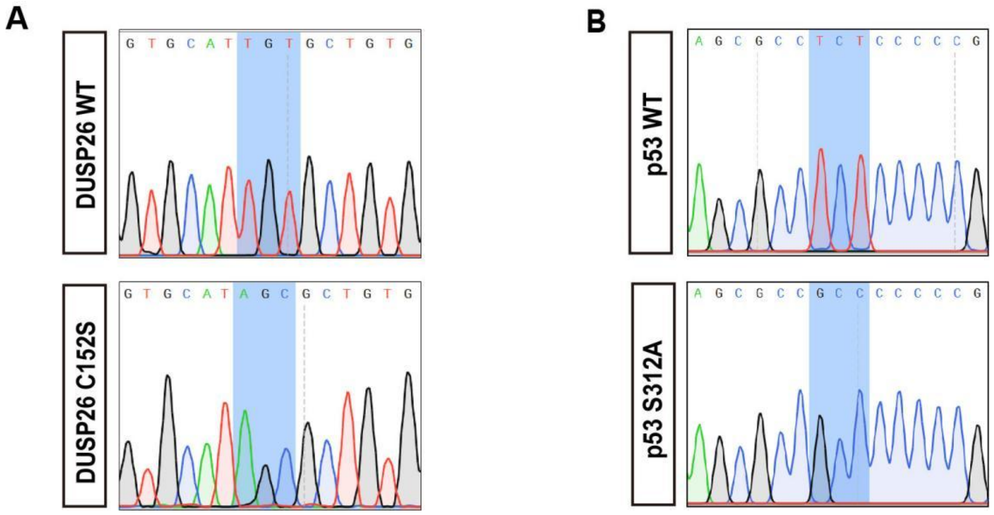

Supplementary Figure 3. Validation of DUSP26 catalytic mutant and p53 S312A plasmids.

(A) Site-directed mutagenesis of the catalytic cysteine at position 152 (C152) to serine (S) was performed to reduce DUSP26 phosphatase activity.

(B) Sanger sequencing validation of wild-type p53 (p53-WT) and p53 Ser312-to-Ala

(S312A) mutant plasmids.

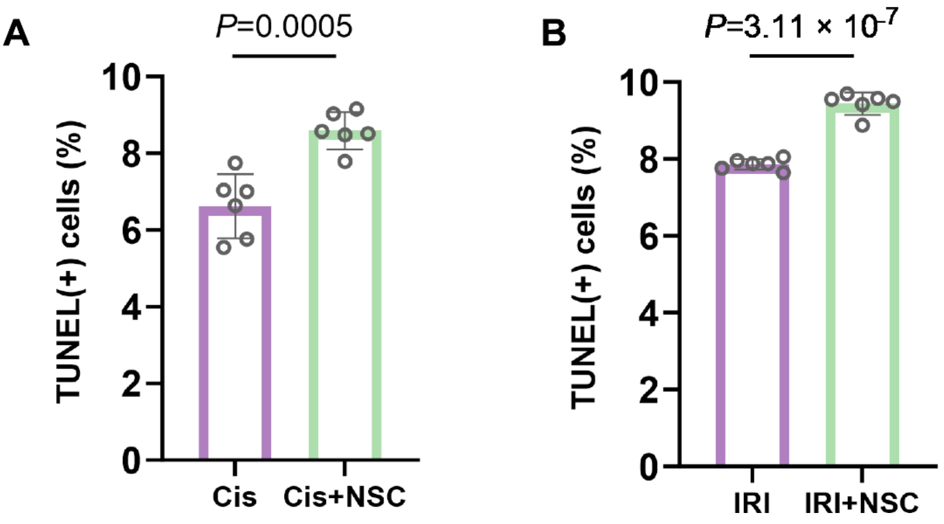

Supplementary Figure 4. Quantification of TUNEL-positive cells in DUSP26-inhibited AKI models.

(A) Quantification of the percentage of TUNEL-positive cells in kidney tissues from mice treated with cisplatin (Cis) or cisplatin plus the DUSP26 inhibitor NSC87877 (Cis+NSC).

(B) Quantification of the percentage of TUNEL-positive cells in kidney tissues from mice subjected to ischemia-reperfusion injury (IRI) or IRI plus NSC87877 (IRI+NSC).

Each dot represents one biologically independent mouse (n = 6 per group). For each mouse, TUNEL-positive nuclei were quantified in 10 randomly selected fields and averaged to yield one value per mouse. Data are mean ± SEM with individual data points overlaid. Statistical significance was assessed by unpaired Student's t-test (two-sided). Exact *P* values are shown in the plots. Source Data are provided as a Source Data file.

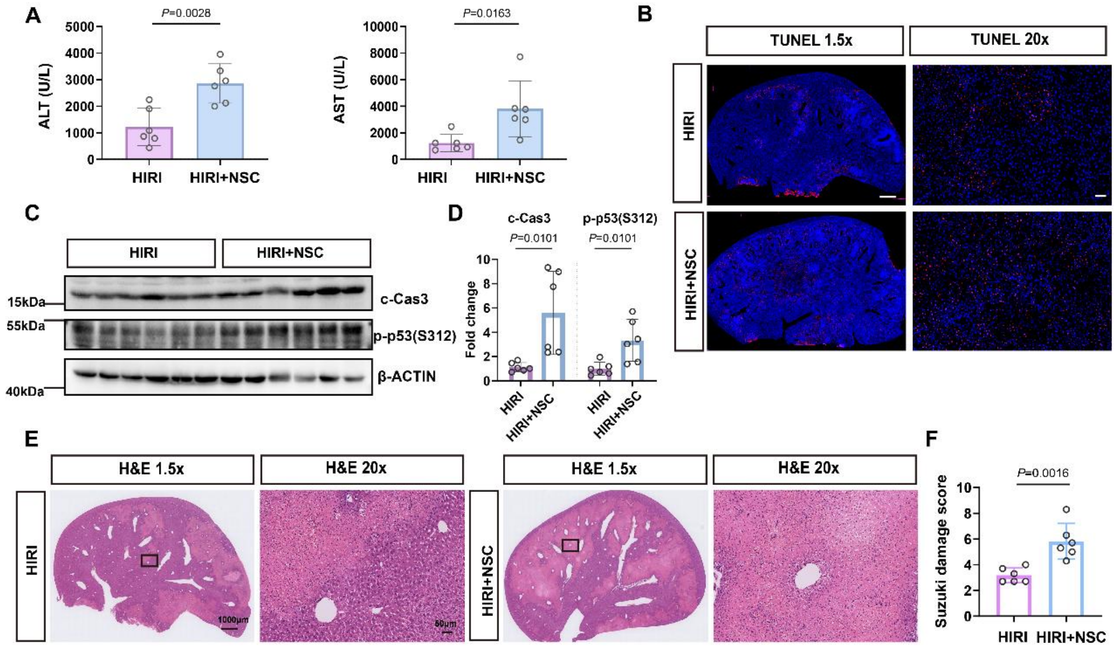

Supplementary Figure 5. NSC87877 exacerbates liver ischemia-reperfusion injury (HIRI).

(A) Experimental scheme: mice were pretreated with NSC87877 and subjected to partial hepatic ischemia (portal triad clamping, 1.5 h) followed by 24 h reperfusion. Serum alanine aminotransferase (ALT) and aspartate aminotransferase (AST) were measured. Each dot represents one biologically independent mouse (n = 6 per group).

(B) Representative TUNEL staining of liver sections from HIRI and HIRI+NSC groups. Scale bars, 1000 μm (1.5×) and 50 μm (20×). Images are representative of biologically independent mice (n = 6 per group) with similar results; two sections per mouse.

(C) Representative immunoblots of cleaved caspase-3 (c-Cas3) and phospho-p53 (Ser312) in liver lysates from HIRI and HIRI+NSC groups (n = 6 biologically independent mice per group with similar results). Molecular weight markers (kDa) are shown.

(D) Quantification of c-Cas3 and phospho-p53 (Ser312) from (C), normalized to the loading control and expressed relative to HIRI. Each dot represents one biologically independent mouse (n = 6 per group).

(E) Representative H&E-stained liver sections from HIRI and HIRI+NSC groups.

Scale bar, 50 µm. Images are representative of biologically independent mice (n = 6 per group) with similar results; two sections per mouse.

(F) Suzuki liver injury score based on sinusoidal congestion, hepatocyte ballooning, and necrosis. For each mouse, 10 randomly selected fields were scored and averaged to yield one value per mouse; each dot represents one biologically independent mouse (n = 6 per group).

Data in (A, D, F) are mean ± SEM with individual data points overlaid. Statistical significance was assessed by unpaired Student's t-test (two-sided). Exact *P* values are shown in the plots. Source data are provided as a Source Data file.

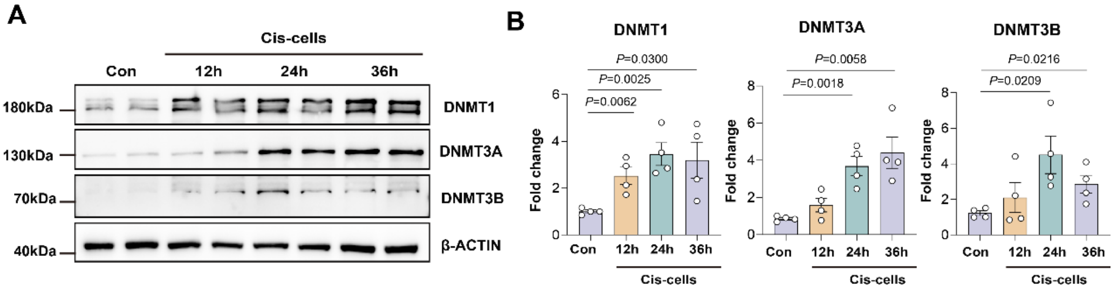

Supplementary Figure 6. Cisplatin treatment induces the expression of DNA methyltransferases (DNMTs) in BUMPT cells.

(A) Representative immunoblot of DNMT1, DNMT3A and DNMT3B in BUMPT cells treated with cisplatin (Cis; 20 µM) for 12, 24 and 36 h; Con, untreated control. β-ACTIN, loading control. Blots are representative of n = 4 biologically independent experiments (independent cell cultures/treatments) with similar results. Molecular weight markers (kDa) are shown.

(B) Densitometric quantification of DNMT1, DNMT3A and DNMT3B from (A), normalized to β-ACTIN and expressed as fold change relative to Con. Each dot represents one biologically independent experiment (n = 4 per group).

Data are mean ± SEM with individual data points overlaid. Statistical significance was assessed by one-way ANOVA with Tukey's multiple-comparisons test (two-sided; adjusted *P* values). Exact *P* values are shown in the plots. Source data are provided as a Source Data file.

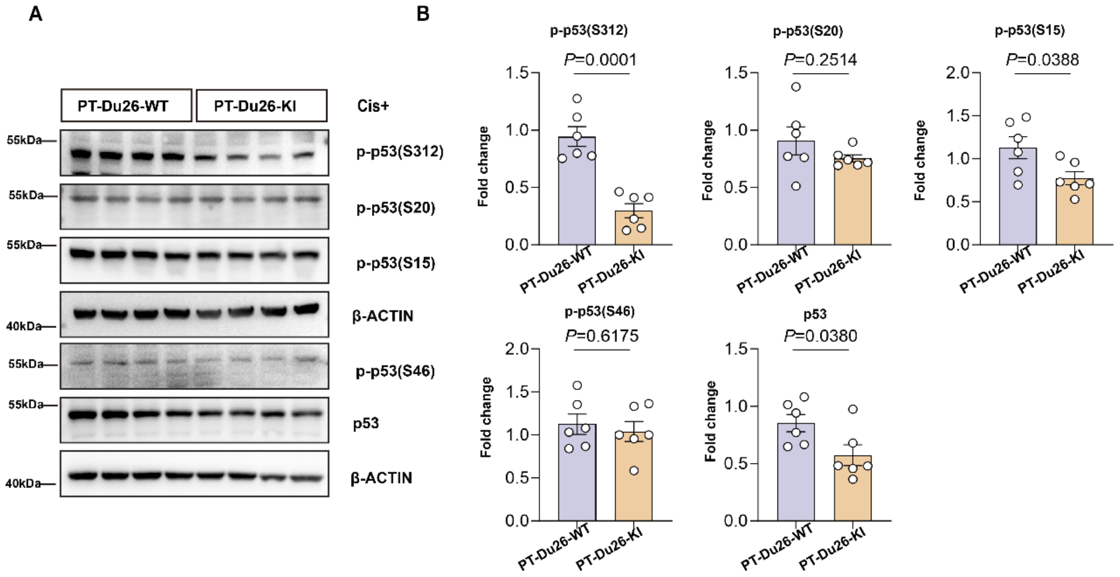

Supplementary Figure 7. DUSP26 overexpression in vivo specifically inhibits p53-Ser312 phosphorylation, but not N-terminal sites, during cisplatin-induced AKI.

(A) Representative immunoblot of phospho-p53 at Ser312, Ser20, Ser15 and Ser46, and total p53 in kidney lysates from PT-Dusp26-WT and PT-Dusp26-KI mice 48 h after cisplatin. β-ACTIN, loading control. Blots are representative of biologically independent mice (n = 6 per group) with similar results and were reproduced in two independent experiments with similar results. Molecular weight markers (kDa) are shown.

(B) Densitometric quantification of p-p53(Ser312), p-p53(Ser20), p-p53(Ser15), p-p53(Ser46) and total p53 from (A), normalized to β-ACTIN and expressed as fold change relative to PT-Dusp26-WT. Each dot represents one biologically independent mouse (n = 6 per group).

Data are mean ± SEM with individual data points overlaid. Statistical significance was assessed by unpaired Student's t-test (two-sided). Exact *P* values are shown in the plots. Source data are provided as a Source Data file.

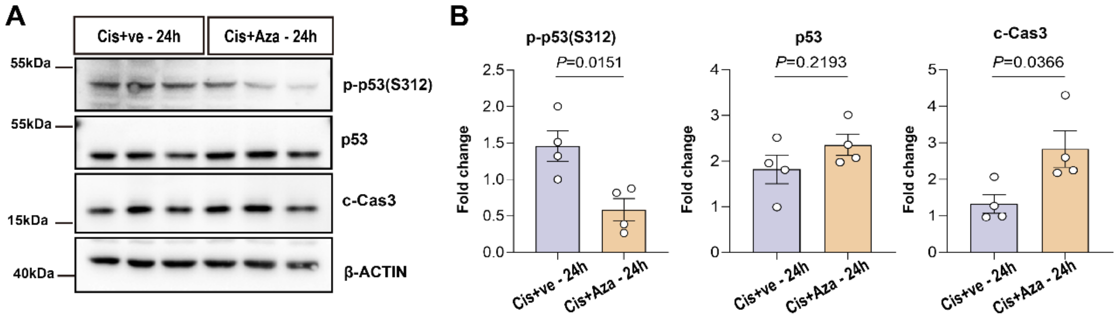

Supplementary Figure 8. 5-Aza treatment paradoxically increases apoptosis despite reducing p-p53(S312) phosphorylation in cisplatin-injured cells.

(A) (A) Representative immunoblot of phospho-p53(Ser312), total p53 and cleaved caspase-3 (c-Cas3) in BUMPT cells treated with cisplatin (Cis) plus vehicle (Ve) or 5-Aza-2′-deoxycytidine (Aza) for 24 h. β-ACTIN, loading control. Blots are representative of biologically independent experiments (n = 4 independent cell cultures/treatments per group) with similar results and were reproduced in two independent experiments with similar results. Molecular weight markers (kDa) are shown.

(B) Densitometric quantification of p-p53(Ser312), total p53 and c-Cas3 from (A), normalized to β-ACTIN and expressed relative to Cis+Ve. Each dot represents one biologically independent experiment (n = 4 per group).

Data are mean ± SEM with individual data points overlaid. Statistical significance was assessed by unpaired Student's t-test (two-sided). Exact *P* values are shown in the plots. Source data are provided as a Source Data file.

Supplementary Table 1. Clinical characteristics, estimated glomerular filtration rate (eGFR), and serum creatinine levels of 8 AKI patients undergoing kidney biopsy.

**Case**	**Age (years)**	**Sex**	**Diagnosis**	**eGFR (ml/min/1.73 m^2)**	**Serum Creatinine (μmol/L)**
1	49	Female	FSGS, ATN	6	625.5
2	17	Male	FSGS, ATN	54.3	162.5

3	60	Female	FSGS, ATN	25.3	182.9
4	48	Female	FSGS, ATN	6.9	491
5	46	Male	MCD, ATN	8.1	500
6	58	Female	MCD, ATN	44.2	113
7	56	Female	MCD, ATN	51.5	93
8	37	Female	IgA nephropathy, ATN	53.5	102

Abbreviations: MCD, minimal change disease; ATN, acute tubular necrosis; FSGS, focal segmental glomerulosclerosis; eGFR, estimated glomerular filtration rate.

Supplementary Table 2. Primer sequences used in this study.

Mouse Primers	Forward (5’-3’)	Reverse (5’-3’)
Dusp26	ATGCCCTCTGTTCACCATCC	CTGTTGTGTGAGGCGTTGAG
Bax	AGGATGCGTCCACCAAGAAGCT	TCCGTGTCCACGTCAGCAATCA
Puma	ACCGCTCCACCTGCCGTCAC	ACGGGCGACTCTAAGTGCTGC
Noxa	TCAGGAAGATCGGAGACAAA	TGAGCACACTCGTCCTTCAA
ChIP Primers		
Dusp26	ATTGGTCTACAGGGCAGCAG	CCCCTGTTTACAGCCCCG
Bax	TGCATACAAGCCTGGTCTGG	TGGAAGTGGTAGCCCATAGC
Puma	GTCTGTGTGTATGCGGGAGA	GCCATCCAGAGCCCTTTCAA
MSP primers		
M	GGTAGTAGTTTTGGCGTGTAC	AAAACACCGTATCACGCAACG
U	GGTAGTAGTTTTGGTGTGTAT	AAAACACCATATCACACAACA
TBS primers		
*Dusp26*_1	TGTAATTTATGAATTTTGTTTGGGAAG	CATAACACRCACATCRAAACAA ATTCCTCTC

Dusp26_2	GTTGTAAATAGGGGYGGGTTGTG	TCTCTACCTCCTAATCRCAAAA ATCTCA
Dusp26_3	GGGGGTYGYGAAAGAGTTATGGGTAT	ACCCRCCACCAAAAACCCTAA

Supplementary fig.2A

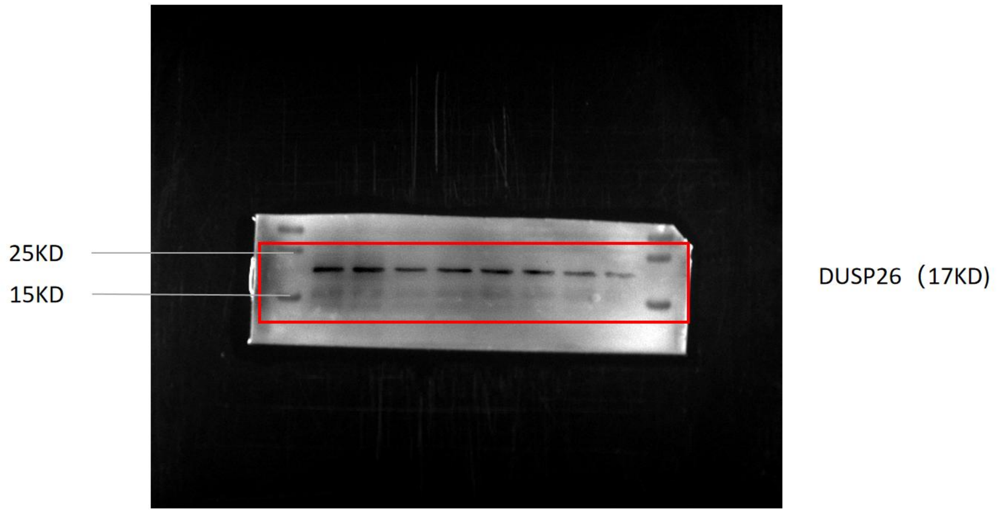

Supplementary fig.2A

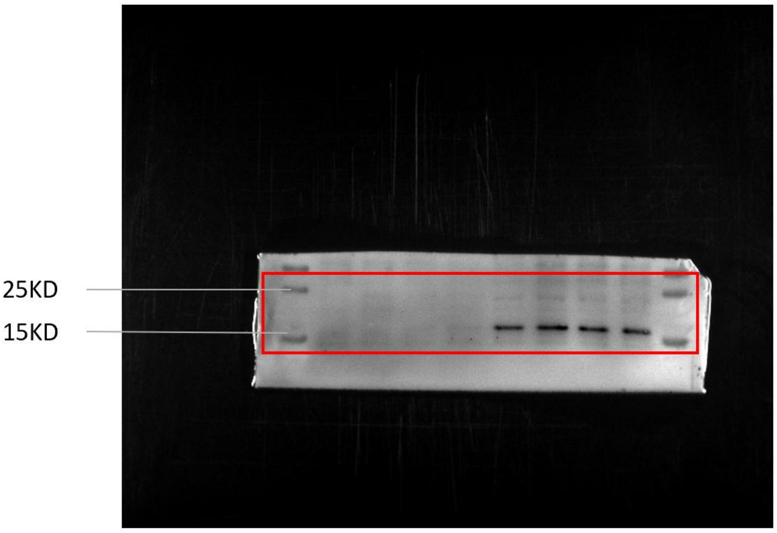

Supplementary fig.2A

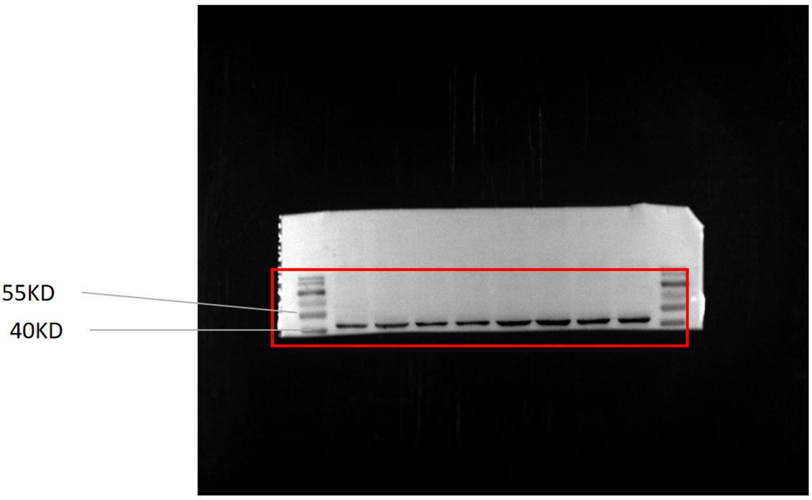

Supplementary fig.2C

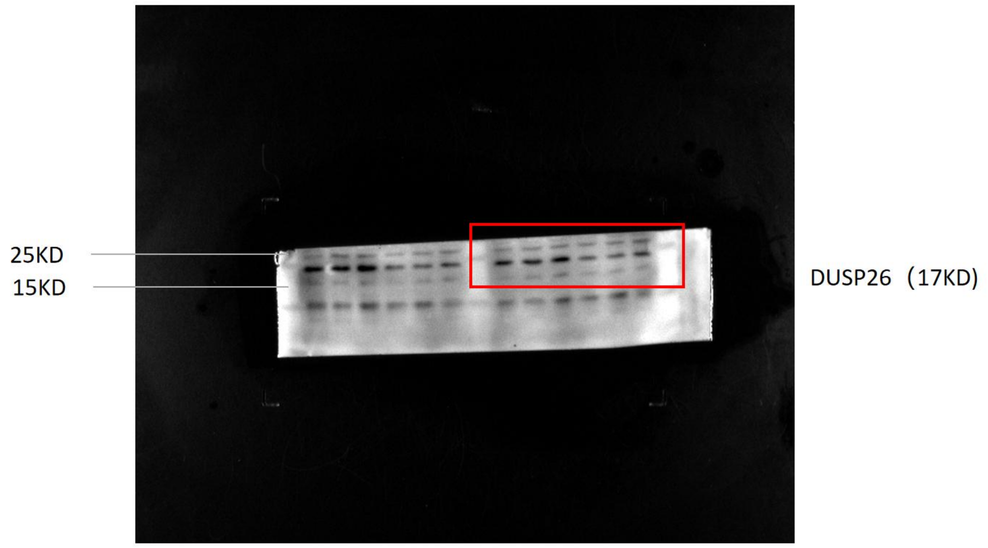

Supplementary fig.2C

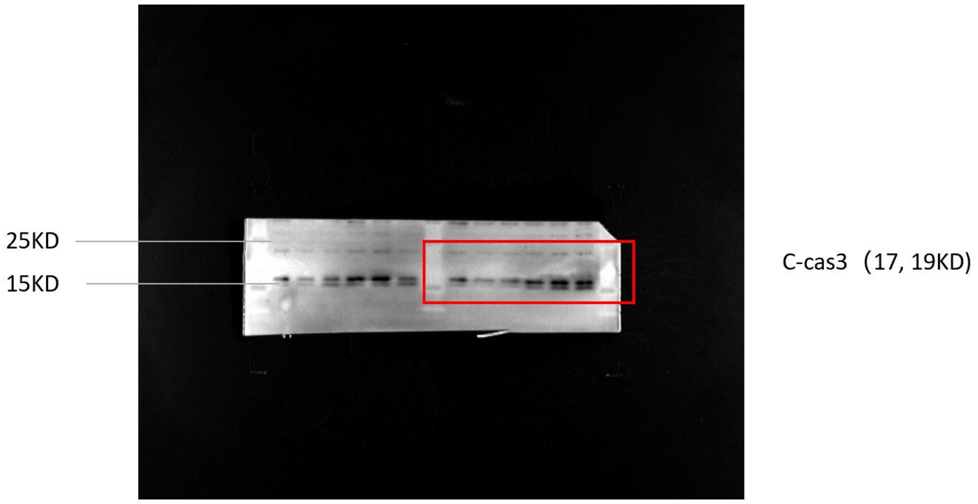

Supplementary fig.2C

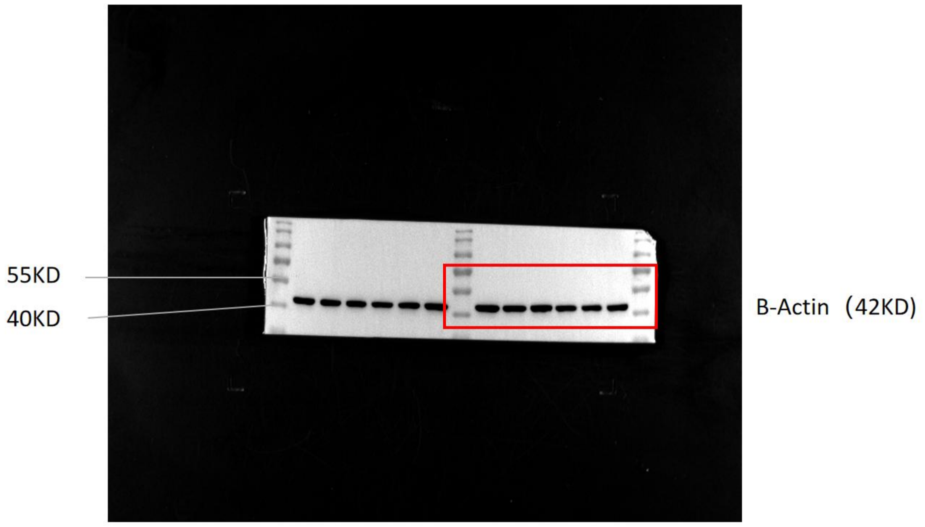

Supplementary fig.5

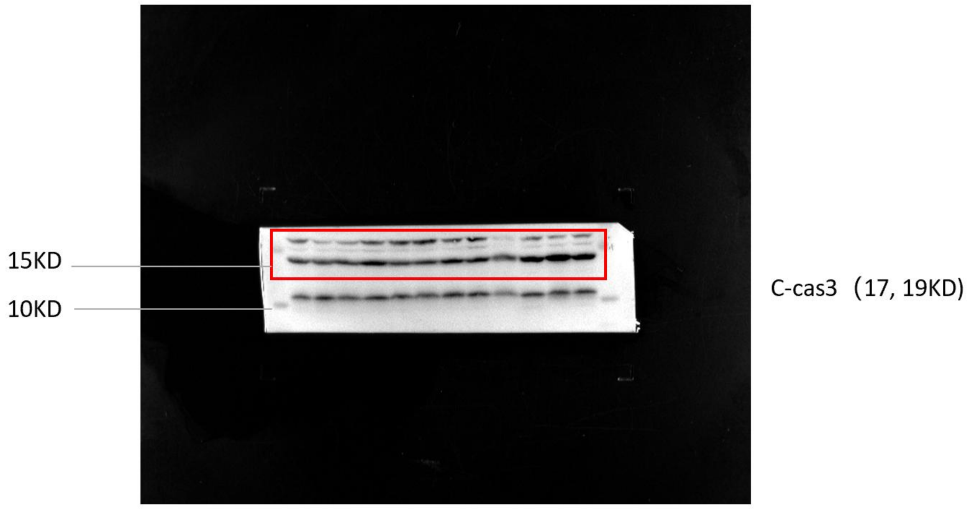

Supplementary fig.5

55KD
40KD

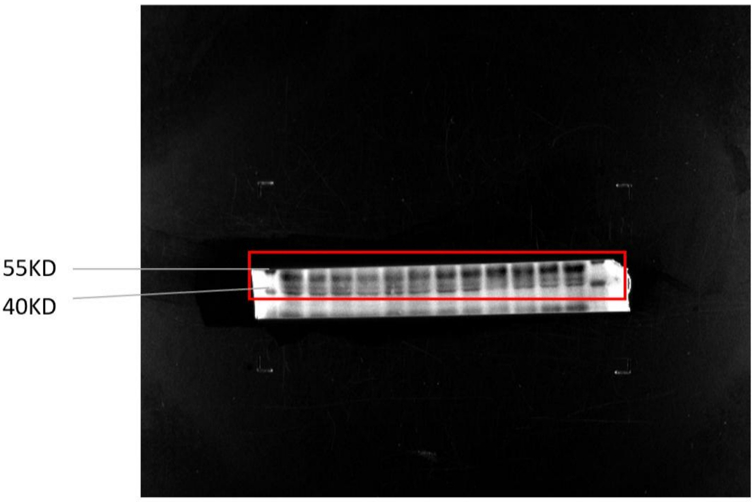

P-p53(S312)（53KD)

Supplementary fig.5

55KD
40KD

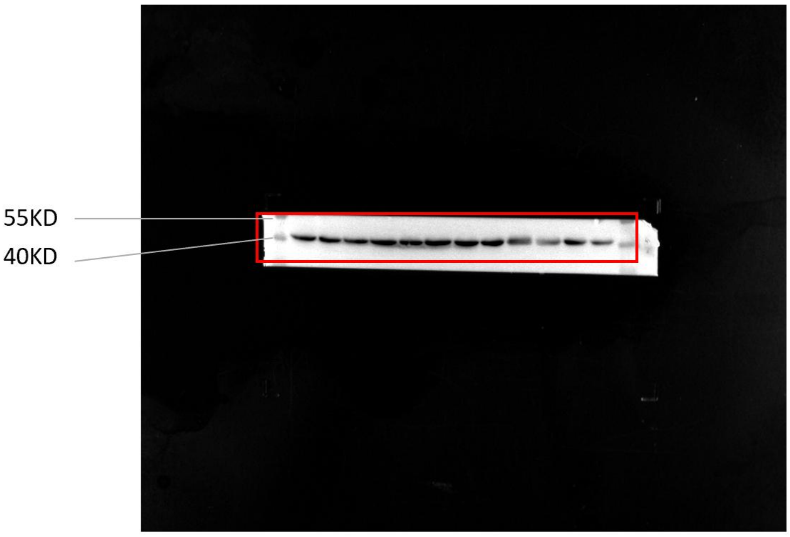

B-Actin（42KD)

Supplementary fig.6

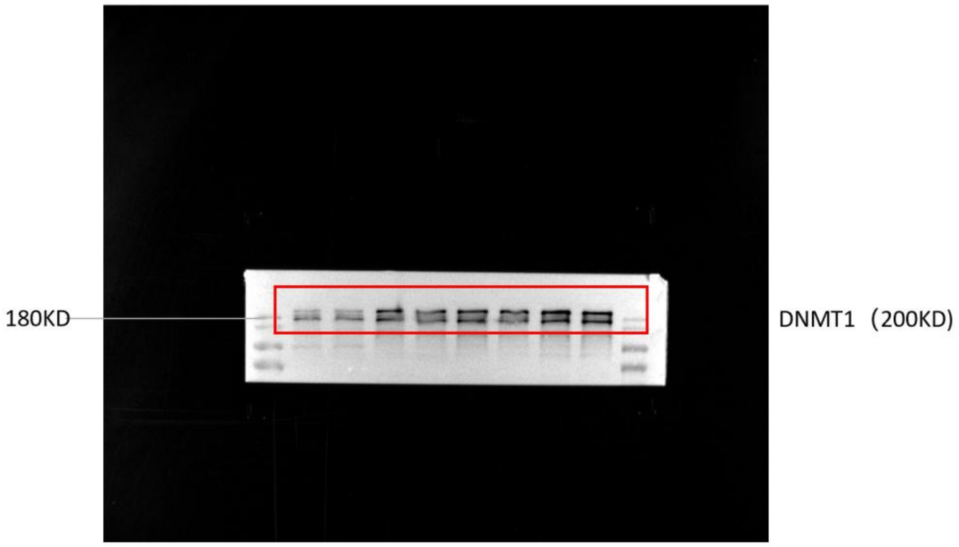

Supplementary fig.6

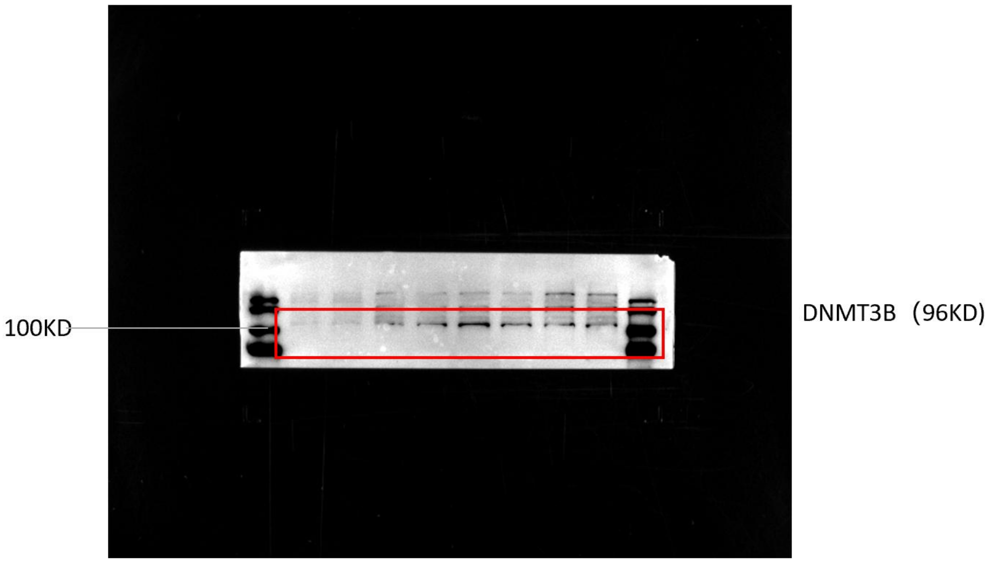

Supplementary fig.6

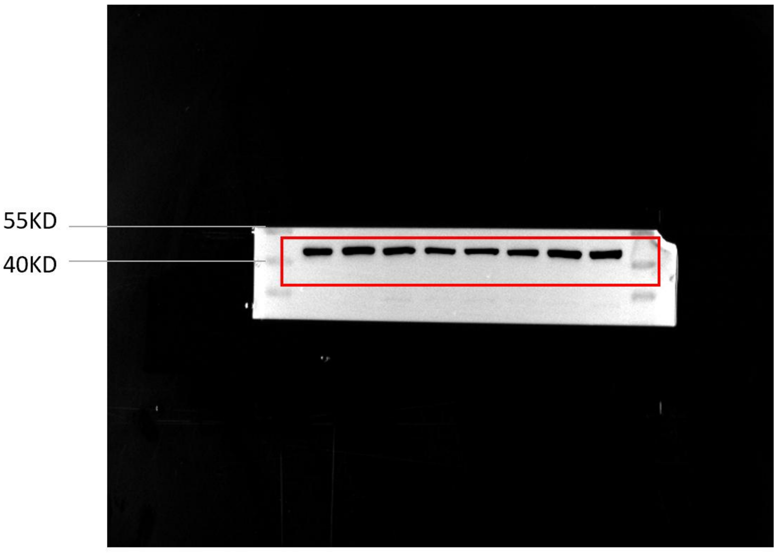

Supplementary fig.6

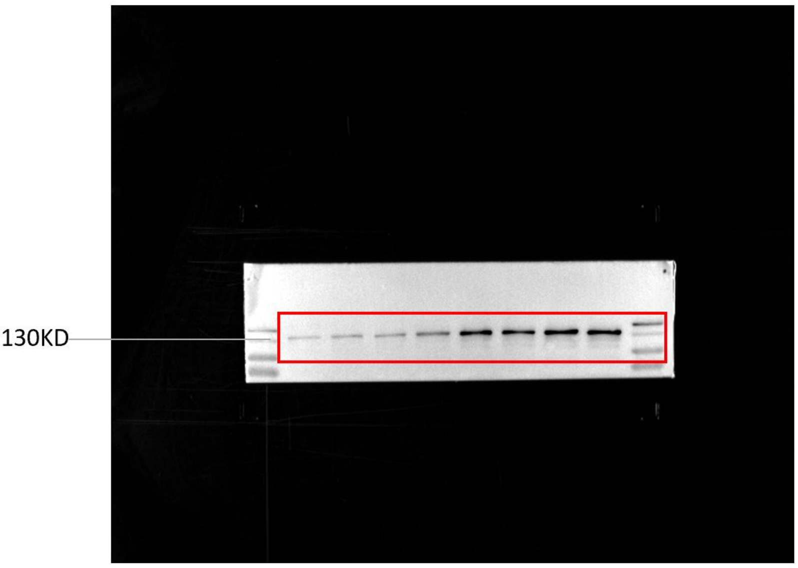

Supplementary fig.7

55KD

40KD

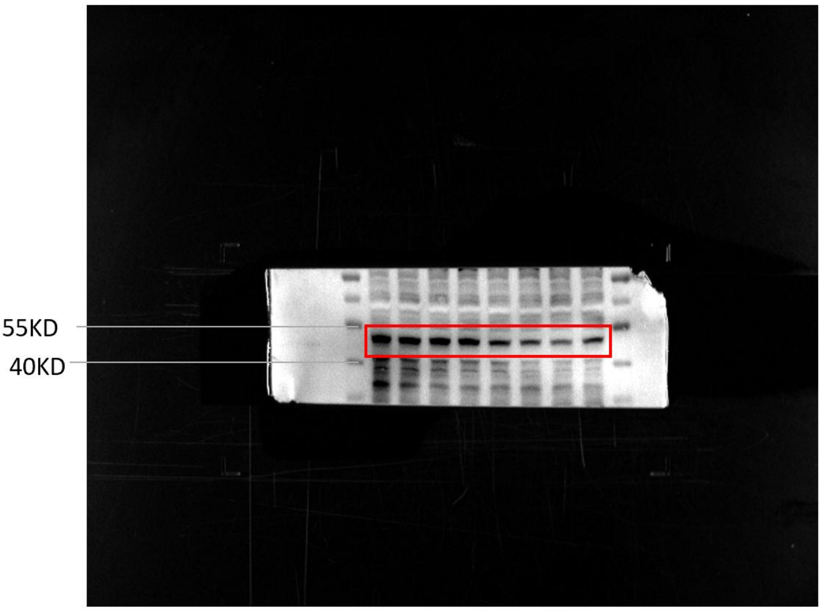

P-p53(s312)

Supplementary fig.7

55KD

40KD

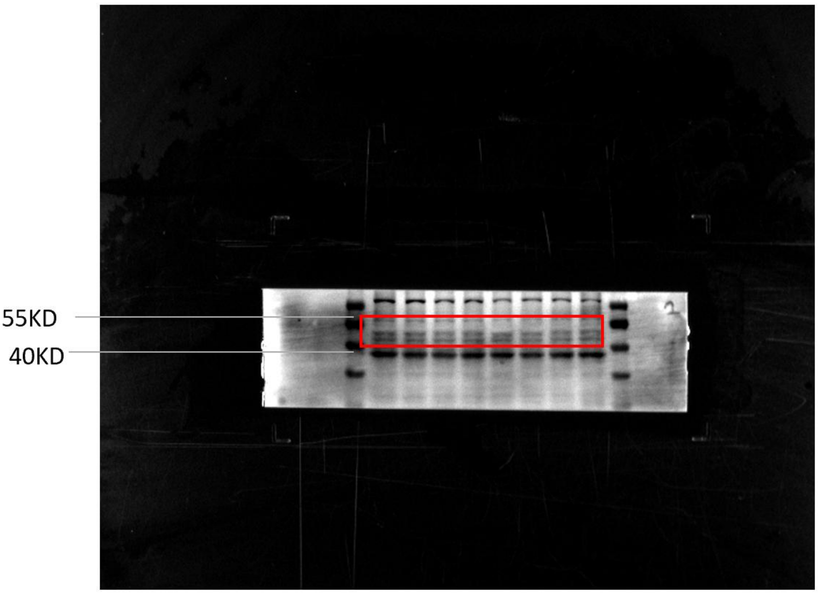

P-p53(s20)

Supplementary fig.7

55KD
40KD

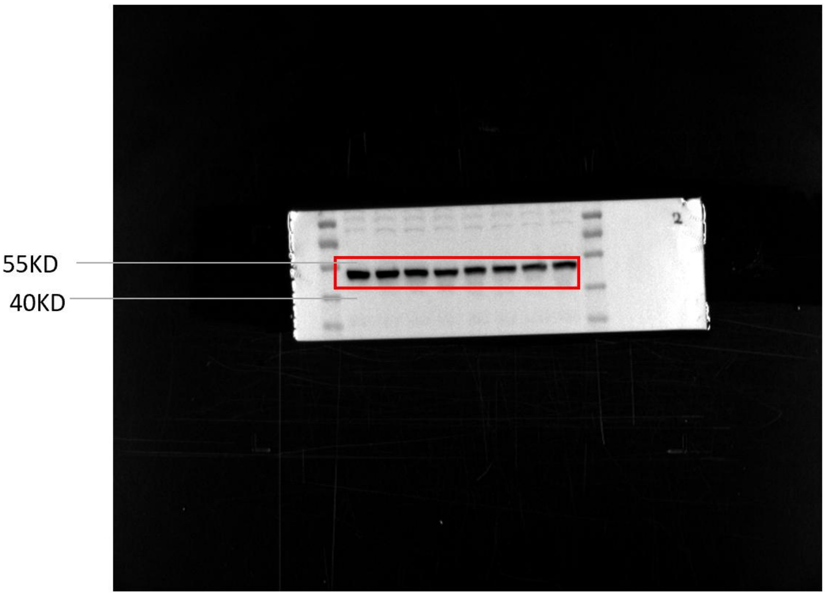

P-p53(s15)

Supplementary fig.7

55KD
40KD

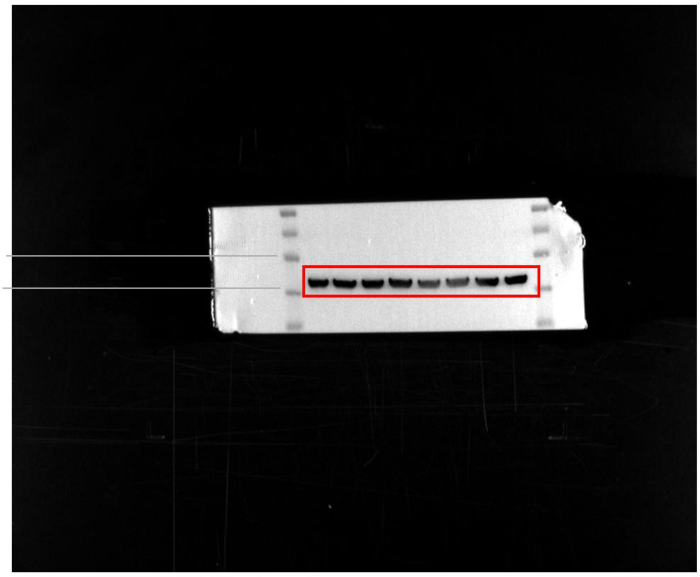

B-Actin（42KD)

Supplementary fig.7

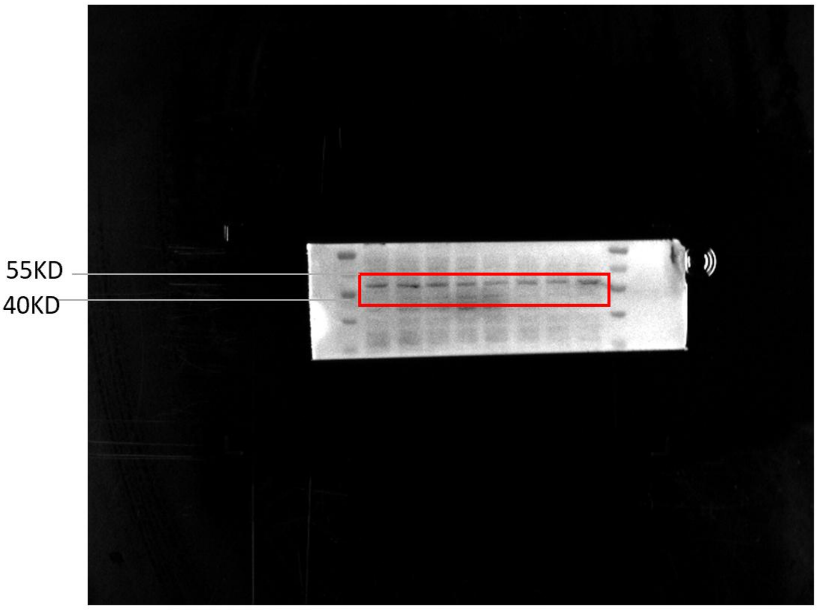

Supplementary fig.7

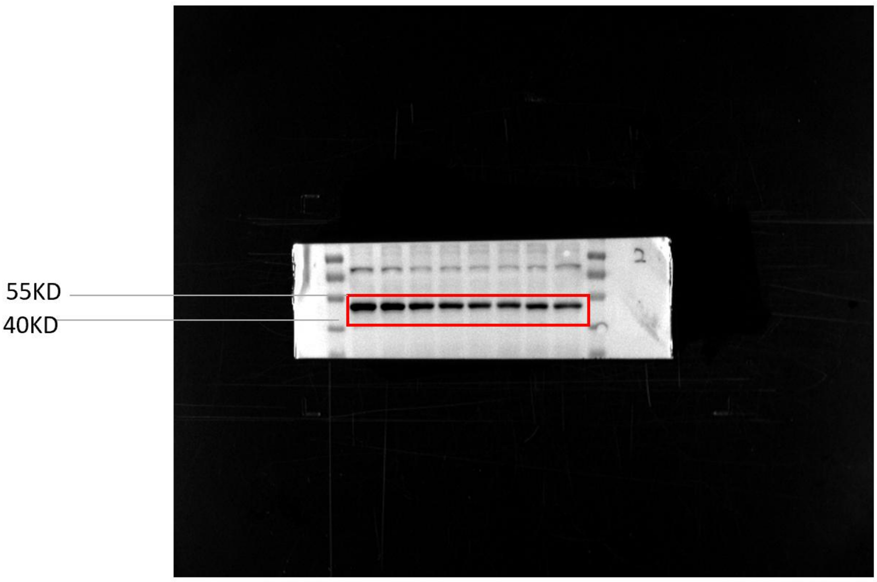

Supplementary fig.7

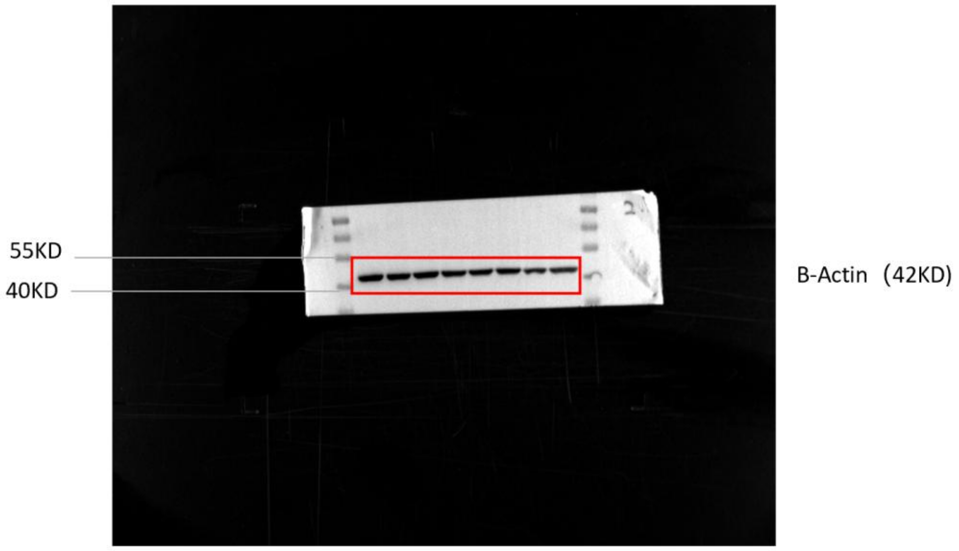

Supplementary fig.8

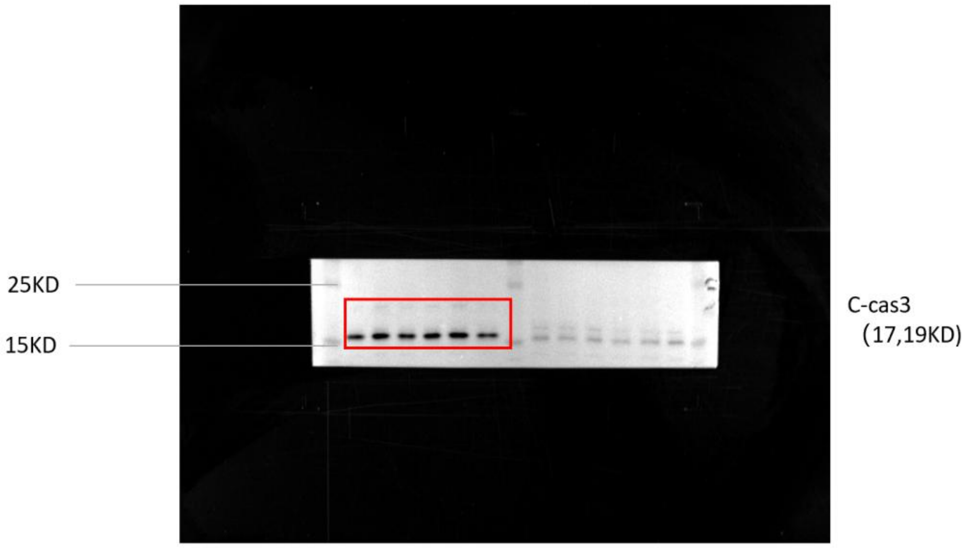

Supplementary fig.8

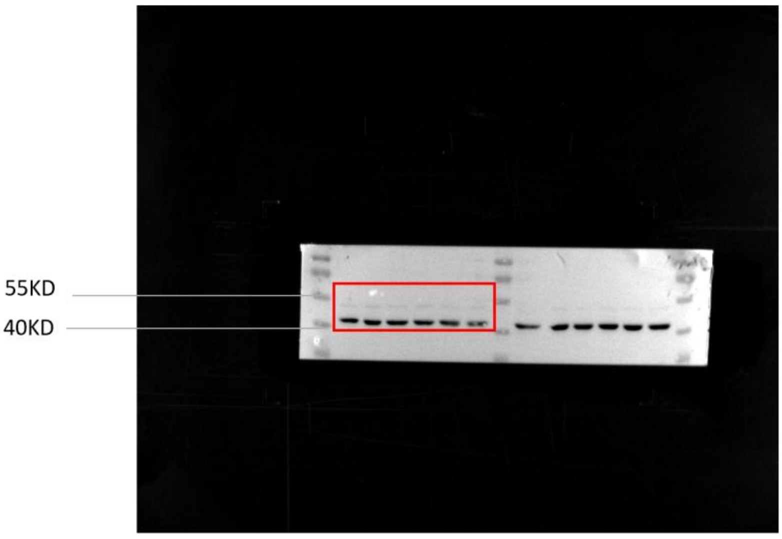

Supplementary fig.8

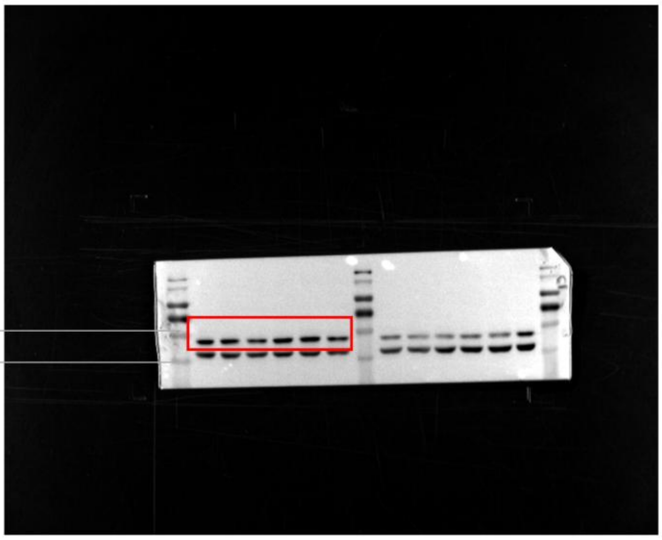

Supplementary fig.8

55KD

40KD

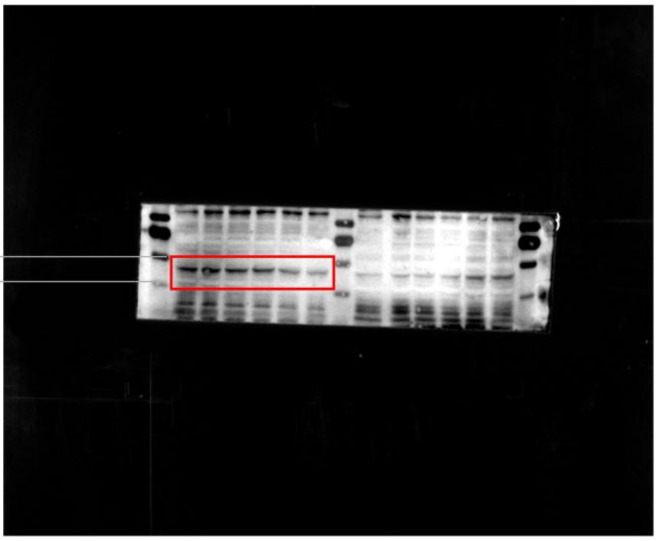

p-p53 s312（53KD）
