# Supplementary material for: DUSP26 protects against acute kidney injury by dephosphorylating p53 at serine 312
Source: Nat Commun. 2026 Feb 26;17:3208. doi: 10.1038/s41467-026-69688-3 (PMC13056927; doi:10.1038/s41467-026-69688-3)
Supplement: Supplementary file 2 — Reporting summary [file 41467_2026_69688_MOESM2_ESM.pdf]

nature portfolio

Corresponding author(s): Zheng Dong

Last updated by author(s): Jan 27, 2026

Reporting Summary

Nature Portfolio wishes to improve the reproducibility of the work that we publish. This form provides structure for consistency and transparency in reporting. For further information on Nature Portfolio policies, see our Editorial Policies and the Editorial Policy Checklist.

Statistics

For all statistical analyses, confirm that the following items are present in the figure legend, table legend, main text, or Methods section.

n/a	Confirmed	
☐	☒	The exact sample size (*n*) for each experimental group/condition, given as a discrete number and unit of measurement
☐	☒	A statement on whether measurements were taken from distinct samples or whether the same sample was measured repeatedly
☐	☒	The statistical test(s) used AND whether they are one- or two-sided<br>*Only common tests should be described solely by name; describe more complex techniques in the Methods section.*
☒	☐	A description of all covariates tested
☐	☒	A description of any assumptions or corrections, such as tests of normality and adjustment for multiple comparisons
☐	☒	A full description of the statistical parameters including central tendency (e.g. means) or other basic estimates (e.g. regression coefficient) AND variation (e.g. standard deviation) or associated estimates of uncertainty (e.g. confidence intervals)
☐	☒	For null hypothesis testing, the test statistic (e.g. *F*, *t*, *r*) with confidence intervals, effect sizes, degrees of freedom and *P* value noted<br>*Give P values as exact values whenever suitable.*
☒	☐	For Bayesian analysis, information on the choice of priors and Markov chain Monte Carlo settings
☒	☐	For hierarchical and complex designs, identification of the appropriate level for tests and full reporting of outcomes
☐	☒	Estimates of effect sizes (e.g. Cohen's *d*, Pearson's *r*), indicating how they were calculated

Our web collection on statistics for biologists contains articles on many of the points above.

Software and code

Policy information about availability of computer code

Data collection	Images were acquired using ZEN Blue edition (v3.7; Carl Zeiss, Germany) for fluorescence microscopy and CaseViewer (v2.4.0; 3DHISTECH, Hungary) for whole-slide digital scanning. Chemiluminescent western blot signals were captured using Tanon Image Analysis Software (v4.2; Tanon, China). Targeted bisulfite sequencing (TBS) was performed on an Illumina NovaSeq 6000 platform using NovaSeq Control Software (v1.7.5; Illumina, USA). Real-time PCR data were collected using LightCycler 96 Application Software (v1.1; Roche, Germany) and QuantStudio Real-Time PCR Software (v1.3; Applied Biosystems, USA). Mass spectrometry data were acquired on a Q Exactive system using Xcalibur (v4.1; Thermo Fisher Scientific, USA).
Data analysis	Western blot quantification was performed using ImageJ (v1.54g; NIH, USA). Histological and immunofluorescence quantification was conducted using Image-Pro Plus (v6.0; Media Cybernetics, USA). Proteomics raw files were processed with MaxQuant (v1.6.14). TBS data processing and methylation calling were performed using fastp (v0.20.0) and Bismark (v0.22.3). Bioinformatic and machine learning analyses were performed in R (v4.4.0) using the following packages: sva (v3.52.0), limma (v3.60.0), glmnet (v4.1-8), e1071 (v1.7-14), caret (v6.0-94), pROC (v1.18.5), pheatmap (v1.0.12), ggplot2 (v3.5.1), and DSS (v2.43.2). Statistical analyses and graphing were performed using GraphPad Prism (v10.0; GraphPad Software, USA).

For manuscripts utilizing custom algorithms or software that are central to the research but not yet described in published literature, software must be made available to editors and reviewers. We strongly encourage code deposition in a community repository (e.g. GitHub). See the Nature Portfolio guidelines for submitting code & software for further information.

Data

Policy information about availability of data

All manuscripts must include a data availability statement. This statement should provide the following information, where applicable:

- Accession codes, unique identifiers, or web links for publicly available datasets
- A description of any restrictions on data availability
- For clinical datasets or third party data, please ensure that the statement adheres to our policy

Publicly available data were downloaded from the Gene Expression Omnibus (GEO) under accession numbers GSE21374, GSE30718, and GSE43974, which were used for secondary analysis. The mass spectrometry proteomics data have been deposited to the ProteomeXchange Consortium via the PRIDE partner repository with the dataset identifier PXD070789. The bisulfite sequencing data generated in this study have been deposited in the NCBI BioProject database under accession code PRJNA1365351. Source data are provided with this paper.

Research involving human participants, their data, or biological material

Policy information about studies with human participants or human data. See also policy information about sex, gender (identity/presentation), and sexual orientation and race, ethnicity and racism.

Reporting on sex and gender	Sex was considered in the study design. The study involved kidney tissue samples from both male and female participants (detailed in Supplementary Table 1). Sex was determined based on biological attributes recorded in medical records. No gender-based analysis was performed as the study utilized retrospective tissue samples.
Reporting on race, ethnicity, or other socially relevant groupings	Race and ethnicity data were not collected or used as covariates in this study, as the research focused on molecular mechanisms in tissue samples.
Population characteristics	The study included kidney biopsy samples from patients clinically diagnosed with Acute Tubular Necrosis (ATN, n=8) and control samples from nephrectomy specimens (n=7). Detailed population characteristics, including age, sex, and clinical indicators (e.g., serum creatinine), are provided in Supplementary Table 1.
Recruitment	No participants were prospectively recruited. This study involved the secondary use of de-identified archival FFPE sections obtained from clinically indicated diagnostic kidney biopsies (ATN group) or nephrectomy specimens (controls from the uninvolved pole of localized renal tumors). Specimens were identified retrospectively from the pathology archive of Second Xiangya hospital based on histological diagnosis.
Ethics oversight	The study protocol was approved by the Institutional Ethics Committee of the Second Xiangya Hospital, Central South University (Approval No. 2020YFC2005004).Written informed consent was obtained from all participants for the use and publication of de-identified clinical information and archival tissue sections; for participants under 18 years of age, consent was obtained from a parent or legal guardian in accordance with institutional guidelines. A blank copy of the informed consent form is provided as a supplementary file with the final submission.

Note that full information on the approval of the study protocol must also be provided in the manuscript.

Field-specific reporting

Please select the one below that is the best fit for your research. If you are not sure, read the appropriate sections before making your selection.

☒ Life sciences ☐ Behavioural & social sciences ☐ Ecological, evolutionary & environmental sciences

For a reference copy of the document with all sections, see nature.com/documents/nr-reporting-summary-flat.pdf

Life sciences study design

All studies must disclose on these points even when the disclosure is negative.

Sample size	No statistical methods were used to predetermine sample size. Sample sizes were chosen based on experimental feasibility, expected effect sizes from pilot experiments, and common practice in the field. For human analyses, each individual sample represented one biological replicate (Control n=7; ATN n=8). For animal studies, each mouse was considered one biological replicate; group sizes were selected to allow robust detection of biologically meaningful differences and are consistent with prior AKI/IRI studies, with exact n for each experiment reported in the figure legends and Source Data. For in vitro experiments, at least three independent biological replicates (independent cell preparations and treatments on different days) were performed for quantitative assays (e.g., WB and qPCR), and results were reproducible across repeats as indicated.
Data exclusions	Generally no data were excluded from the analyses unless technical failures occurred (e.g., sample contamination, instrument error) or when mice were dropped out for analyses due to accidental deaths of the mice.
Replication	Experiments were repeated with the same conditions and obtained similar results. The number of repeats were indicated in figure legends.

Randomization	Experimental groups were randomly assigned. For in vitro studies, the experiment was carried out strictly in accordance with the single variable principle. Treatment groups were divided randomly and equally.
Blinding	Blinding was not feasible due to obvious phenotypic differences between groups, but results were validated by an independent observer. Tubular injury of mice was scored in a blinded manner based on the percentage of injured tubules in the renal cortex.

Reporting for specific materials, systems and methods

We require information from authors about some types of materials, experimental systems and methods used in many studies. Here, indicate whether each material, system or method listed is relevant to your study. If you are not sure if a list item applies to your research, read the appropriate section before selecting a response.

Materials & experimental systems

n/a	Involved in the study
☐	☒ Antibodies
☐	☒ Eukaryotic cell lines
☒	☐ Palaeontology and archaeology
☐	☒ Animals and other organisms
☐	☒ Clinical data
☒	☐ Dual use research of concern
☒	☐ Plants

Methods

n/a	Involved in the study
☒	☐ ChIP-seq
☒	☐ Flow cytometry
☒	☐ MRI-based neuroimaging

Antibodies

Antibodies used	Commercial antibodies:DUSP26 (Invitrogen; Cat# PA5-22013; rabbit polyclonal; IF 1:100; WB 1:1000).KIM-1 (R&D Systems; Cat# AF1817; goat polyclonal; IF 1:200; WB 1:1000).NGAL (R&D Systems; Cat# AF1857; goat polyclonal; WB 1:1000).p53 (Cell Signaling Technology; Cat# 2524S [WB] / 2524 [ChIP]; Clone 1C12; mouse monoclonal; WB 1:1000; ChIP 0.8 µg/well).p-p53 (S15) (Cell Signaling Technology; Cat# 9284S; rabbit polyclonal; WB 1:1000).p-p53 (S20) (Cell Signaling Technology; Cat# 9287T; rabbit polyclonal; WB 1:1000).p-p53 (S46) (Proteintech; Cat# 28960; rabbit polyclonal; WB 1:1000).Cleaved caspase-3 (Cell Signaling Technology; Cat# 9664S; Clone 5A1E; rabbit monoclonal; WB 1:1000).GAPDH (Proteintech; Cat# 10494; rabbit polyclonal; WB 1:1000).beta-actin (Proteintech; Cat# 66009; mouse monoclonal; WB 1:1000).DNMT1 (Cell Signaling Technology; Cat# 5032; rabbit monoclonal; WB 1:1000; ChIP 0.8 µg/well).DNMT3A (abcam; Cat# ab188470; rabbit monoclonal; WB 1:1000).DNMT3B (abcam; Cat# ab2851; rabbit polyclonal; WB 1:1000).DNMT3A (Cell Signaling Technology; Cat# 9768; rabbit monoclonal; ChIP 0.8 µg/well).DNMT3B (Cell Signaling Technology; Cat# 44145; rabbit monoclonal; ChIP 0.8 µg/well).FLAG (Sigma-Aldrich; Cat# 102698217; mouse monoclonal; WB 1:1000).Normal mouse IgG (Cell Signaling Technology; Cat# 5415; mouse monoclonal; ChIP 0.8 µg/well).Normal rabbit IgG (Cell Signaling Technology; Cat# 2729; rabbit polyclonal; ChIP 0.8 µg/well).Lotus tetragonolobus lectin (LTL) (Vector Laboratories; Cat# RFL-1321; lectin; IF 1:100).Custom antibody:Anti–p-p53 (Ser312) (rabbit; generated by Hangzhou HuaAn Biotechnology Co., Ltd.; affinity-purified; used in WB as described in Methods).
Validation	Commercial antibodies: Commercial primary antibodies were validated by the respective manufacturers for the listed applications. Specificity was further supported in this study by expected staining patterns (IF) and/or detection of bands at the expected molecular weights (WB) together with appropriate positive/negative controls. In particular, cleaved caspase-3 (CST; Cat# 9664S; Clone 5A1E) was manufacturer-validated for Western blotting and used here as a standard apoptosis marker.Custom antibody validation: The phospho-specific antibody against p53(Ser312) was generated using a synthetic phosphopeptide immunogen and purified by affinity chromatography (phosphopeptide enrichment followed by depletion using the non-phosphorylated peptide). Antibody titer and phospho-specificity were initially assessed by ELISA against phosphorylated versus non-phosphorylated peptides. Biological specificity was validated in vivo: the p-p53(S312) signal was detected in cisplatin-injured kidneys expressing wild-type p53 but was absent in kidneys expressing the non-phosphorylatable p53-S312A mutant, confirming site-specific recognition of phosphorylated Ser312.

Eukaryotic cell lines

Policy information about cell lines and Sex and Gender in Research

Cell line source(s)	Mouse proximal tubular epithelial cells (BUMPT), originally established by Dr. Lieberthal at Boston University.
Authentication	None of the cell lines used were authenticated.
Mycoplasma contamination	All cell lines tested negative for mycoplasma contamination.
Commonly misidentified lines (See ICLAC register)	No commonly misidentified cell lines were used in the study.

Animals and other research organisms

Policy information about studies involving animals; ARRIVE guidelines recommended for reporting animal research, and Sex and Gender in Research

Laboratory animals	Species/Strain/Source: Mus musculus. C57BL/6J mice were purchased from SJA Laboratory Animal Corporation. PT-Du26-KI mice (on C57BL/6J background) were generated by crossing Rosa26-LSL-DUSP26 mice (Shanghai Model Organisms Center) with Pepck-Cre mice (provided by Dr. Volker Haase). p53-KO mice (on C57BL/6J background) were obtained from Cyagen Bioscience.Sex: Only male mice were used to minimize the confounding effects of estrogen on AKI susceptibility.Age/Weight: 8–10 weeks old (22–25 g) at the start of all experiments.Housing: SPF conditions, 21–26°C, 40–60% humidity, 12 h dark/12 h light cycle.
Wild animals	No wild animals were used in this study.
Reporting on sex	To minimize the potential confounding effects of estrogen, which is known to confer protection against acute kidney injury (AKI) in females.
Field-collected samples	No field-collected samples were used in this study.
Ethics oversight	All animal procedures complied with relevant ethical regulations and the NIH Guide for the Care and Use of Laboratory Animals and were approved by the Institutional Animal Care and Use Committee (IACUC) of the Second Xiangya Hospital, Central South University (Approval No. 2022418).

Note that full information on the approval of the study protocol must also be provided in the manuscript.

Clinical data

Policy information about clinical studies

All manuscripts should comply with the ICMJE guidelines for publication of clinical research and a completed CONSORT checklist must be included with all submissions.

Clinical trial registration	Not applicable. This study is a retrospective analysis of human tissue samples and does not qualify as a clinical trial.
Study protocol	Not applicable.
Data collection	Kidney tissue samples and clinical data were collected retrospectively from the Department of Nephrology, Second Xiangya Hospital, Central South University.
Outcomes	The primary outcome was the expression level of DUSP26 in renal tubular cells, which was assessed by immunofluorescence staining and correlated with the clinical/pathological indices (ATN score and eGFR).

Plants

Seed stocks	Not applicable. This study did not involve any plant materials or seeds.
Novel plant genotypes	Not applicable. This study did not involve any plant materials or seeds.
Authentication	Not applicable. This study did not involve any plant materials or seeds.
